# Experimental Study on the Stiffness of Steel Beam-to-Upright Connections for Storage Racking Systems

**DOI:** 10.3390/ma13132949

**Published:** 2020-07-01

**Authors:** Florin Dumbrava, Camelia Cerbu

**Affiliations:** 1 Department of Mechanical Engineering, Faculty of Mechanical Engineering, Transilvania University of Brasov, B-dul Eroilor, No. 29, 500036 Brasov, Romania; florin.dumbrava@unitbv.ro; 2 Product Development Department, S.C. Dexion Storage Solutions S.R.L, Str. Campului Nr. 1A, 505400 Rasnov, Romania

**Keywords:** storage systems, stiffness, tab connector, flexural test, capable design moment

## Abstract

The aspects regarding the stiffness of the connections between the beams that support the storage pallets and the uprights is very important in the analysis of the displacements and stresses in the storage racking systems. The main purpose of this paper is to study the effects of both upright thickness and tab connector type on the rotational stiffness and on the capable bending moment of the connection. For this purpose, a number of 18 different groups of beam-connector-upright assemblies are prepared by combining three types of beams (different sizes of the box cross section), three kinds of uprights profiles (with a different thickness of the section walls), and two types of connectors (four-tab connectors and five-tab connectors). Flexural tests were carried out on 101 assemblies. For the assemblies containing the uprights having the thickness of 1.5 mm, the five-tab connector leads to a higher value of the capable moment and higher rotational stiffness than similar assemblies with four-tab connectors. A contrary phenomenon happens in case of the assemblies containing the upright profiles having a thickness of 2.0 mm regarding the capable design moment. It is shown how the safety coefficient of connection depends on both the rotational stiffness and capable bending moment.

## 1. Introduction

In recent years, there has been a significant increase in consumption, which has led to an increase of producing the storage racking systems. The products are often stored in pallet raking systems in super-markets or in warehouses of products or raw materials within factories.

Pallet racking systems are self-supporting structures that need to support considerable vertical weights. They are generally made of two main types of components: frames and beams. The frames are made of thin-walled upright profiles that have perforations in their length through which the joints with the beams are usually realized by using metallic connectors with tabs [1,2]. Such connectors are welded to both ends of the beams. Horizontal and diagonal braces welded between the uprights ensure the stability of the frame in a cross-aisle direction. The beams provide the stability in down-aisle direction and the stiffness of the connections is an important characteristic of the storage racking systems from this point of view.

Besides metallic connectors with tabs, there are other types of connections used in constructions, which include metallic connectors with screws or bolts [3], wood-steel-wood connections whose external parts are made of wood and the internal part is made of steel [4], and connectors with tabs combined with additionally fixing using bolts [5,6]. The last type of connections are commonly known as speed lock connections [7].

In a simple way, the connections used between beams and uprights are either hinged (pin-connected) or rigid connections. In practice, for almost all connections, even for those considered as rigid connections, there may be certain rotations that influence the internal forces, especially the bending moments. Not all the connections that are considered as being rigid connections are completely rigid in reality. On the other hand, the connections considered as being hinged are not perfectly hinged because, in practice, frictions can take place and the friction influences the rotation of the connection. Therefore, estimating the stiffness of the connections becomes a necessity, especially for the connections used for the storage racking structures.

Following the emergence of EN 1993-1-3, Eurocode 3 [8], a classification of the connections used between beams and uprights was made, based on their corresponding stiffness. The connections that are investigated within this research fall into the category of those considered as being semi-rigid connections. The stiffness of such connections is experimentally determined, according to the EN 15512 standard [9]. It is essential to understand that is crucial to determine the rotational stiffness of the connections and the capable bending moment because, otherwise, the racking storage systems do not work safety.

The literature is devoid of publications on the behavior in bending tests, of the beam-to-upright connections used in storage racking systems, and results related to the effect of the geometry of different components (beam, upright profile, and beam-end connector) on the connection stiffness and capable moment. Some recent studies [2,10] on the beam-to-upright connections reported the effects of both depth of the beam and type of the tab connection on the connection stiffness and on the failure modes of the connections with tabs in bending tests. It was shown that the common failure modes are tearing of the uprights near the slots for tabs and cracking of the tabs of the connectors [2]. No remarks were made about the failure modes of the beams in bending tests of the beam-to-upright connections. These papers did not make a comparison of the failure modes depending on the number of the tabs for each type of the connector involved. The authors showed the effects of the cross section shapes of the upright profiles on the connections’ stiffness.

From a theoretical point-of-view, some researchers proposed in recent published works [11,12,13] the mechanical model, which includes five basic deformable components (tab in bending, wall of the upright profile in bearing and bending, tab connector in bending and shear) of the beam-to-upright connections in order to predict the initial rotational stiffness of the connections.

Regarding the beams used for storage racking systems, because these are cold-formed steel structural elements with thin-walls, distortional buckling failures may occur in bending and the effects of the shearing force must be accurately evaluated in bending, especially in case of the thin-walled elements having opened a cross section [14,15]. An interesting study [16] presents the results concerning the elastic shear buckling loads and ultimate strength by using numerical simulations of the stresses and strains in case of the finite element models of cold-formed steel channels with slotted webs subjected to shear.

Another critical issue of the metallic storage structures is related to the fire design methodologies so that all thin-walled structural elements to keep the load-bearing capacity as long as possible in the event of the accidental fire. In practice, in order to improve the load-bearing capacity in fire conditions, there are some types of passive or active protection systems [17]. Passive protection systems generally refer to special materials, manufacturing, or surface coating technologies in order to delay the spread of fire [4]. However, in the case of storage systems for supermarkets or warehouses, it usually uses active fire protection systems, which include automatic fire detection and extinguishing equipment (for example, a network of water pipes that are equipped with discharge nozzles called sprinklers) [17]. Regarding the cold-formed steel beams with opened and closed cross sections, a unique fire design methodology validated by experimental tests, was developed in Reference [18]. Experimental and numerical results concerning the mechanical behavior of the cold-formed steel columns subjected in fire conditions are presented in Reference [19]. Regarding the connections, there are numerical models used to predict the load-bearing capacity of the connections with and without passive protection. This is similar to the numerical model for wood-steel-wood connections [4].

Since the main target of the researchers from the industry of the storage racking systems is to increase the rotational stiffness of the beam-to-upright connections, some innovative beam-end connectors with tabs having one additional bolt fixed in the side part of the upright [1] or two additional bolts fixed in the front part of the upright [3,6] were proposed and investigated in monotonic and cyclic bending tests. However, it is known that the increase of the rotational stiffness of the beam-to-upright connections leads to the rise of the bending moment at the beam ends when the beams are mechanically loaded in storage systems. It follows that the beam-to-uprights connections should be evaluated by taking into account the ratio between the capable bending moment and the real value of the bending moment. Moreover, the external force is a distributed force applied on the length of the beam in a real case of loading while the force is applied to the free end of the beam in the bending tests used in research on beam-to-upright connections, according to EN 15512 [9] and MH16.1 [20].

In this context, in the present research, the stiffness and capable moment is experimentally determined in bending tests carried-out on 18 different groups of beam-connector-upright assemblies prepared by combining three types of beams (different sizes of the box cross section), three kinds of uprights profiles (with different thickness of the section wall), and two types of connectors (four-tab connector and five-tab connector). This work is a part of the report research on the mechanical behavior of the thin-walled metal parts used in the racking storage systems subjected to bending and buckling [21].

The main purpose of this research to analyze the effects of the connector type with tabs and the effects of the dimensions of the assembled elements (beams and uprights) used in the racking storage systems on both the rotational stiffness and the capable bending moment at failure of that connection in order to predict the safety coefficients of the beam-to-upright connections. For this purpose, the following objectives are established: (i) testing of different groups of assemblies between the beams and the uprights by using two types of connectors (with four or five tabs), (ii) comparison of the stiffness of the connections in function of the type of the connector, (iii) analysis of the effects of the wall thickness of the upright profile and of the size of a beam cross section on the rotational stiffness and on the capable moment at failure for all beam-to-upright connections involved, (iv) comparison of estimated safety coefficients for all beam-to-upright connections tested in order to predict the best type of connection for each group containing beams of the same size for the case of the beam loaded by the distributed weight of the pallets, (v) comparison of the estimated maximum deflections for beams with the same type of semi-rigid connections at both ends by taking into account the stiffness experimentally obtained.

The authors also aim to use the results obtained experimentally by focusing on the stiffness of the connections in order to compare the maximum deflections of the beams having such semi-rigid connectors at both ends with the maximum deflections computed for the beams pin-connected or with rigid connectors at both ends. Bending moments developed at the midpoint of the beam and at the ends of the beam are also evaluated for different boundary conditions.

## 2. Materials and Methods

### 2.1. Beam-to-Upright Connections Tested

**Figure 1:**
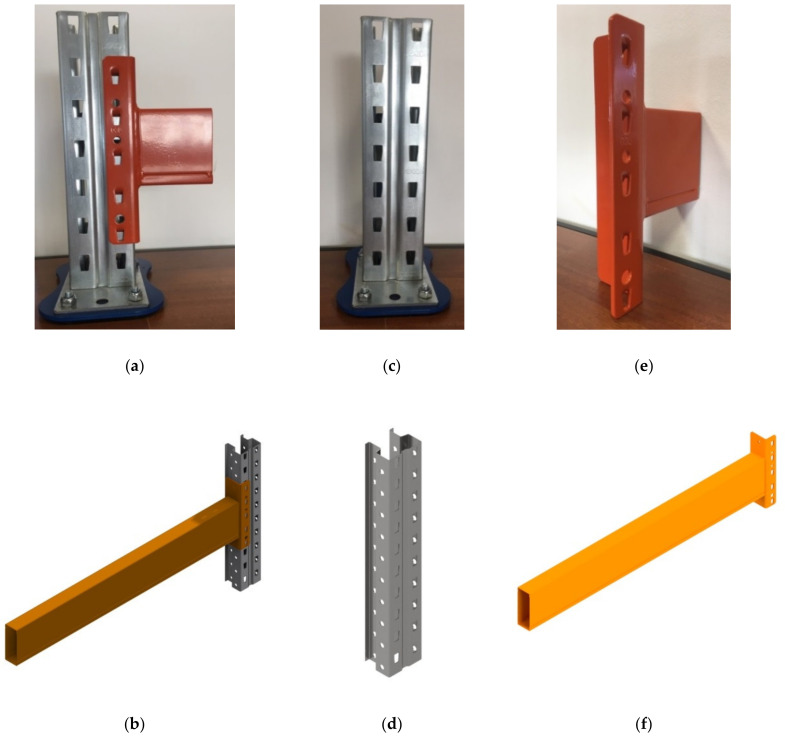
Main elements of the connections tested: (**a**,**b**) upright-connector-beam assembly, (**c**,**d**) upright, (**e**,**f**) beam with welded beam end connector.

The main structural elements of such a semi-rigid joint used for the pallet racking structures are shown in Figure 1.

**Figure 2:**
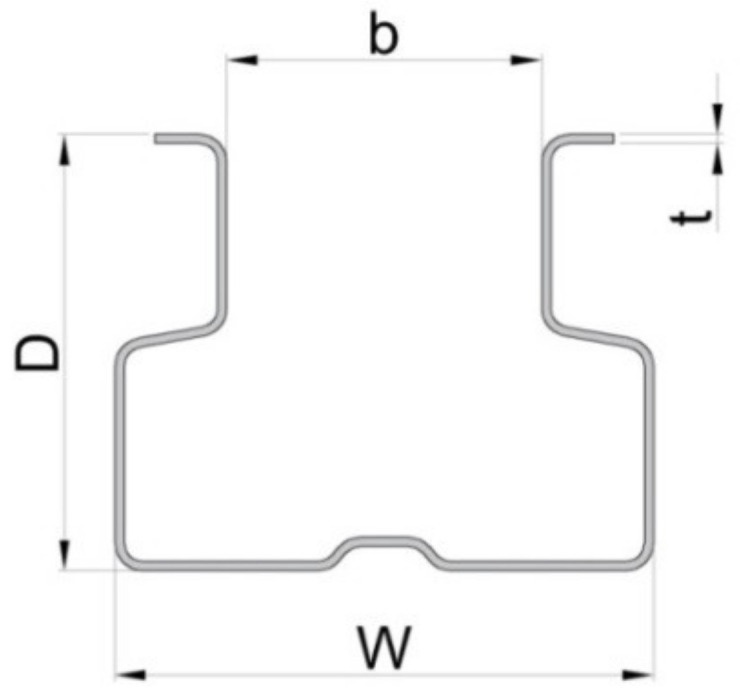
Cross section of the upright profile (dimensions D, W, b, t are given in Table 1).

The upright (Figure 1c,d) is a cold-formed omega-profile made of a thin steel sheet (Figure 2). The upright profile has perforations along its length that facilitate the connection with the beam end connectors welded at the beam end. The main dimensions of the cross sections of the uprights involved in this research are shown in Table 1.

**Figure 3:**
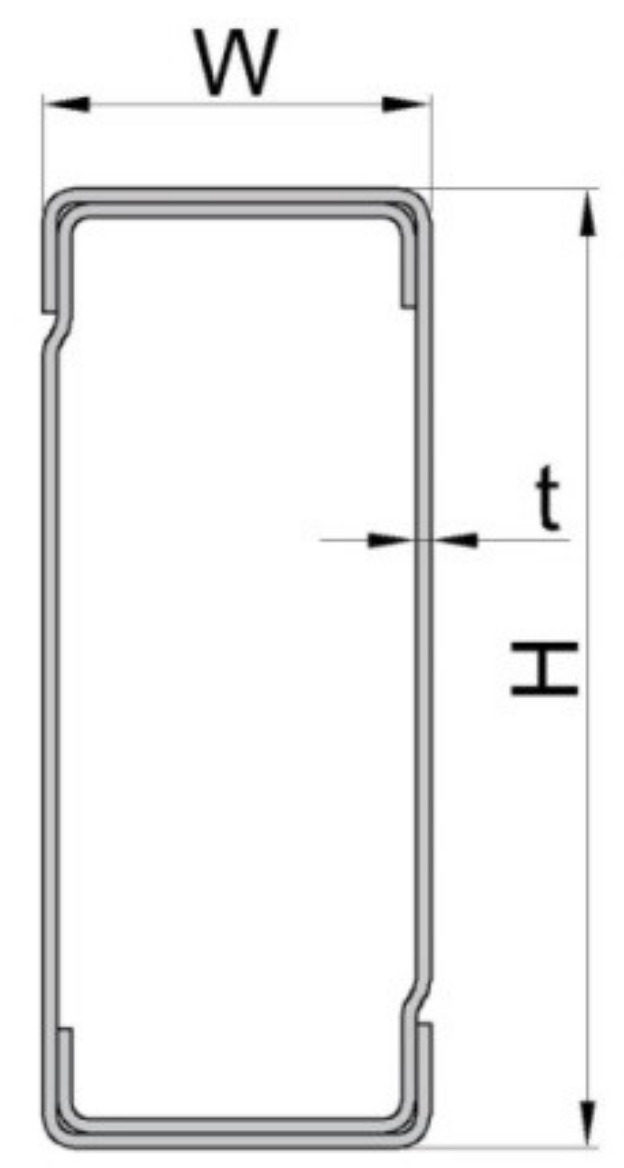
Shape and dimensions of the cross-sections of the beams (dimensions H, W, t are given in Table 2).

Beams having a box cross section (Figure 1e,f) are manufactured by using two C-profiles assembled together in a rectangular shape (Figure 3). The connectors are welded at both ends of the beam and those connectors are used to hook the beam by perforations of the upright. The main dimensions of the cross sections of the beams involved in this study are shown in Table 2.

**Figure 4:**
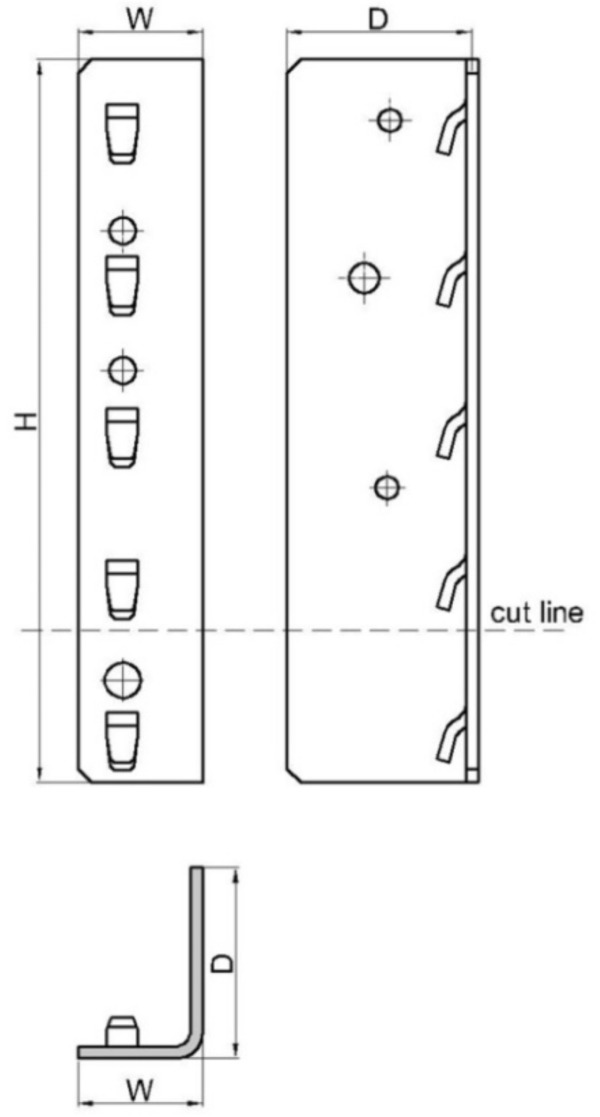
Shape and dimensions of the beam end connectors involved in the research (dimensions H, W, D are given in Table 3).

In Table 3, the overall dimensions (Figure 4) are shown as well as the designation codes corresponding to both types of beam end connectors used in this research.

In Table 4, all upright-connector-beam assemblies tested in bending are shown in this research in order to determine the stiffness of those connections. The corresponding identification codes are also shown in Table 4. The main components of each assembly are: upright, beam, and beam and connector. Three types of upright profiles were used, which have the same geometry of the cross-section (Figure 2) but have different thicknesses of the profile wall (Table 1). Three types of box beams (Figure 3) are used whose dimensions are given in Table 2. For each type of upright used in an assembly, two types of connectors were considered, which include a four-tab connector and a five-tab connector. Lastly, six different types of upright-connector-beam assemblies were tested for each beam type (Table 4). Multiple individual tests were performed for each type of assembly (last column of Table 4). Lastly, 101 individual tests were carried out.

### 2.2. Work Methods

#### 2.2.1. Tensile Tests

The component parts (upright, beam, and beam end connector) are made of steel. Tensile tests were carried out for the tensile specimens cut from each component part of the assemblies involved in order to experimentally obtain the following characteristics of the materials: yield stress, ultimate stress, and elongation until maximum force. The material properties are used in data processing obtained in bending tests of the connections in accordance with EN 15512 standard [9].

**Figure 5:**
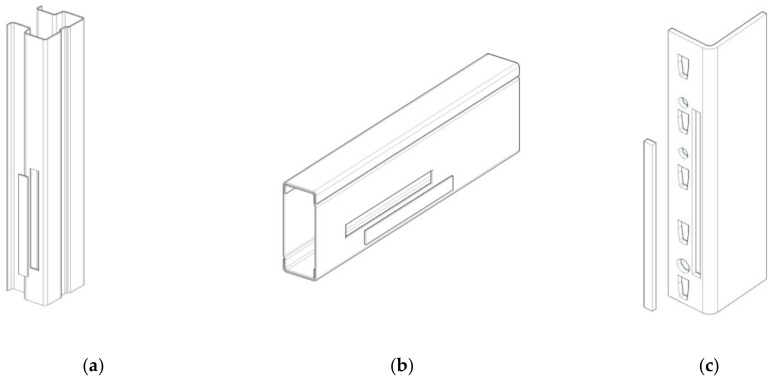
Locations from where we have cut the samples to make tensile tests: (**a**) from upright, (**b**) from beam, and (**c**) from beam end connector.

Tensile specimens were cut from each component part (upright, beam end connector, and beam), as shown in Figure 5.

Universal testing machine INSTRON 3369 (INSTRON, Norwood, MA, USA) with digital controls and a maximum force of 50 kN was used in tensile tests. The tensile testing machine pulls the sample clamped at both ends and records the tensile force related to the elongation of the tensile specimen until it ruptures. By using the force-elongation curve, the stress-strain curve is obtained and we get the yield stress and ultimate stress. In tensile tests, the strain rate was equal to 0.00025 per second until the strain ε = 0.003 and then the strain rate equaled 0.0067 per second until to failure in accordance with the standard EN ISO 6892 [22].

#### 2.2.2. Bending Test of the Connections

**Figure 6:**
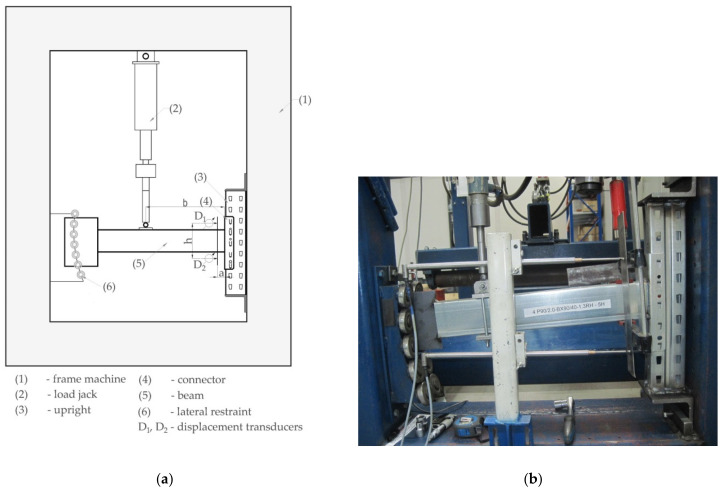
Bending test of the connection according to EN 15512 [9]: (**a**) scheme of the test stand and (**b**) photo of the experimental setup.

Tests have been carried out according to the standard EN 15512 [9]. The scheme of loading used in the bending test of the connection and the photo of the experimental setup are shown in Figure 6.

In accordance with Figure 6 and EN 15512 [9], a short upright cut between two sets of perforations was connected to a very stiff testing frame in two points apart at the distance g = 470 mm from each other. A short beam with a length of 650 mm is connected to the upright region by means of the connector. Sideways movement and twisting of the beam were prevented. The beam is able to move freely in the vertical direction of the load by guiding the free end vertically because a steel plate welded at the free end of the beam is sliding between the bearings. The vertical force was applied at distance b = 400 mm from the face of the upright region by using the force transducer of type S-E-G Instruments (S-E-G Instruments AB, Bromma, Sweden) whose accuracy is 0.001 kN and maximum force is 12.5 kN.

Rotation angle θ of the beam end at the connection with the upright was measured by using the displacement transducers whose rods are in permanent contact with the plate fixed to the beam close to the connector so that the distance is equal to 50 mm (Figure 6a). The displacement transducers of type WA-100 (manufactured by HBM–Hottinger Baldwin Messtechnik Gmbh, Darmstadt, Germany) may record displacements with a size less than 100 mm having the accuracy of 0.001 mm.

The force transducer and both displacement transducers are connected to the QuantumX MX840 Universal Measuring Amplifier (manufactured by Hottinger HBM, Darmstadt, Germany) that transmits the data to Easy Catman software (version 3.1, Hottinger Baldwin Messtechnick Gmbh, Darmstadt, Germany) on the computer. The data acquisition device QuantumX MX840 (Hottinger Baldwin Messtechnick Gmbh, Darmstadt, Germany) works at a frequency of 19.2 kHz.

In the beginning of the bending test, an initial force of approximately 10% of the maximum failure load is applied and, then, the vertical force is increased gradually until failure occurs. Tests were repeated identically for all the upright-connector-beam assemblies involved in this study (Table 4). A minimum of five tests were made for each kind of upright-connector-beam assembly shown in Table 4. In this way, the scattering rate of the results is also analyzed.

The value of the external force F acquired by the force transducer is used to compute the bending moment M by using Equation (1).

(1) M=Fb,

in which b is the dimension shown in Figure 6a.

The deflections D_1_ and D_2_ acquired by the displacement transducers (Figure 6a) are used to compute the rotation angle θ of the beam end at the connection by using Equation (2).

(2) $$\;\theta =\left(D_{2}-D_{1}\right)/h$$

in which h is the distance between the rods of the displacement transducers (Figure 6a). In Equation (2), the deflection D_1_ has a positive value while the deflection D_2_ is a negative value. The rotation angle θ of the beam end computed with Equation (2) is expressed in radians.

The failure mode of the component parts (beam end connector, upright) was also noted in the testing report in the case of each bending test carried out.

The bending moment M and rotation angle θ experimentally obtained were corrected in accordance with the standard EN 15512 [9]. Therefore, the correction procedure is described below.

The corrections are required because there are variations of the yield stress of the material corresponding to each component part (upright, beam, connector) and variations of the thickness corresponding to those components. As a result, the correction factor C_m_ was computed by using Equation (3) and C_m_ must be less than or equal to 1, according to EN 15512 [9].

(3) $$C_{m}={\left[{\left(\frac{f_{y}}{f_{t}}\right)}^{\alpha }\frac{t}{t_{t}}\right]}_{\max}{\mathrm{and}\;C}_{m}\leq 1,$$

where f_t_ is the yield stress obtained by testing the tensile specimens, f_y_ is the nominal yield stress and the values are given in standard EN 10346 [23], t_t_ is the measured thickness for the tensile specimen, t is the design thickness, α = 0 when f_y_ ≥ f_t_, and α = 1 when f_y_ < f_t_.

To make the correction for a moment-rotation curve (M–θ), some steps should be covered in accordance with the EN 15512 standard [9]. First, for each bending test of a connection, the moment-rotation curve (M–θ) is plotted and the slope of the curve K_0_ is measured at the origin. Then, the elastic rotation M/K_0_ is subtracted from the measured rotation θ to obtain the plastic rotation θ_p_ by using Equation (4), according to EN 15512 [9].

(4) $$\theta_{p}=\theta -M/K_{0}.$$

The corrected moment M_n_ is computed by using Equation (5).

(5) $$M_{n}=M\cdot C,$$

where C is another correction factor related to C_m_ whose value is computed by using Equation (6) and must be less than or equal to 1, according to EN 15512 [9].

(6) $$C=0.15+C_{m}\leq 1.$$

The corrected value of the elastic rotation θ_e_ is computed by using Equation (7).

(7) $$\theta_{e}=M_{n}/K_{0}$$

and the corrected rotation angle θ_n_ is computed by using Equation (8), according to EN 15512 [9].

(8) $$\theta_{n}=\theta_{p}+\theta_{e}=\theta_{p}+M_{n}/K_{0}.$$

The adjusted moment-rotation curve (M_n_–θ_n_) is plotted and its initial slope K_0_ is the same for the initial moment-rotation curve (M–θ).

**Figure 7:**
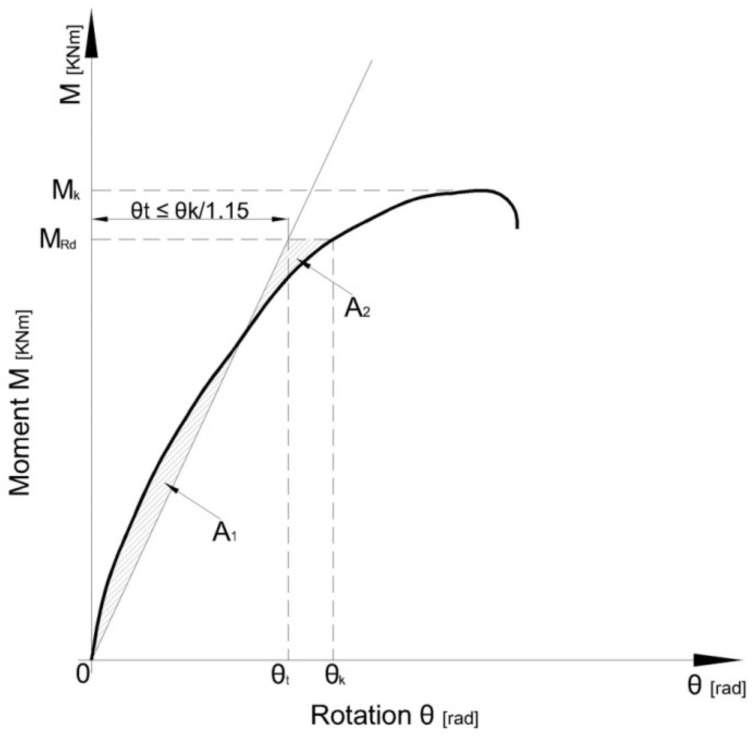
Computing of the rotational stiffness k_ni_, according to EN15512 [9].

The failure moment M_ni_ is considered to be the maximum corrected moment from the adjusted moment-rotation curve (M_n_–θ_n_) shown in Figure 7, which is plotted for each upright-connector-beam assembly. The subscript i of the failure moment M_ni_ represents the number of the test with i = 1,n, where n is the number of tests corresponding to each upright-connector-beam assembly. The moment M_m_ is the mean value of failure moments M_ni_ and it is computed with Equation (9), according to EN 15512 [9].

(9) $$M_{m}=\frac{1}{n}\sum_{i=1}^{n}M_{\mathrm{ni}}.$$

The standard deviation of the adjusted test results denoted with s is computed with Equation (10), according to EN 15512 [9].

(10) $$\;s=\sqrt[{}]{\frac{1}{n-1}\overset{n}{\underset{i=1}{\sum }}{\left(M_{\mathrm{ni}}-M_{m}\right)}^{2}}.$$

For each upright and connector assembly, the characteristic failure moment M_k_ is the characteristic failure moment computed by Equation (11), according to EN 15512 [9].

(11) $$M_{k}=M_{m}-{\mathrm{sK}}_{s},.$$

where K_s_ is the coefficient based on a 95% fractile at a confidence level of 75% in accordance with EN 15512 [9].

The design moment for the connection is denoted by M_Rd_ and is given by Equation (12) [9].

(12) $$M_{\mathrm{Rd}}=\eta \frac{M_{k}}{\gamma_{M}},$$

where γ_M_ is a partial safety factor for the connection and γ_M_ = 1.1, according to EN 15512 [9]. η is the variable moment reduction factor selected by the designer so that η ≤ 1.

The rotational stiffness k_ni_ (i = 1,n) of the connection is obtained as the slope of a line through the origin, which isolates equal areas (A_1_ and A_2_ in Figure 7) located between that line and the experimental curve below the design moment M_Rd_. The rotational stiffness k_ni_ is computed for each test using Equation (13), according to EN 15512 [9].

(13) $$k_{\mathrm{ni}}=\frac{1.15M_{\mathrm{Rd}}}{\theta_{\mathrm{ki}}},$$

where θ_ki_ (i = 1,n) is the rotation corresponding to the design moment M_Rd_ on the adjusted moment-rotation curve (M_n_–θ_n_) shown in Figure 7.

The design rotational stiffness k_m_ is computed by Equation (14) [9].

(14) $$k_{m}=\frac{1}{n}\sum_{i=1}^{n}k_{\mathrm{ni}}.$$

#### 2.2.3. Theoretical Aspects Regarding the Effects of the Stiffness of Connection on Deflections

In strength calculus of the beams of the racking storage systems, the beam is considered to be subjected to uniformly distributed force q given by the specific weight of the goods on the pallets stored on the shelf. It is considered that the modulus of rigidity EI in bending is considered to be constant along the axis of the beam with the length L. By considering the traditional methods to analyze the beams in a racking storage system for which the connections between beam and upright are considered as pin-connections (hinged), the rotation θ is equal to qL^3^/24EI at the beam end at that connection while the bending moment M_b_ developed at the pin-connection is equal to zero. In case the end connections considered as rigid-like is the fixed support, the rotation θ is equal to zero at that connection while the bending moment M_b_ is equal toqL^2^/12EI.

**Figure 8:**
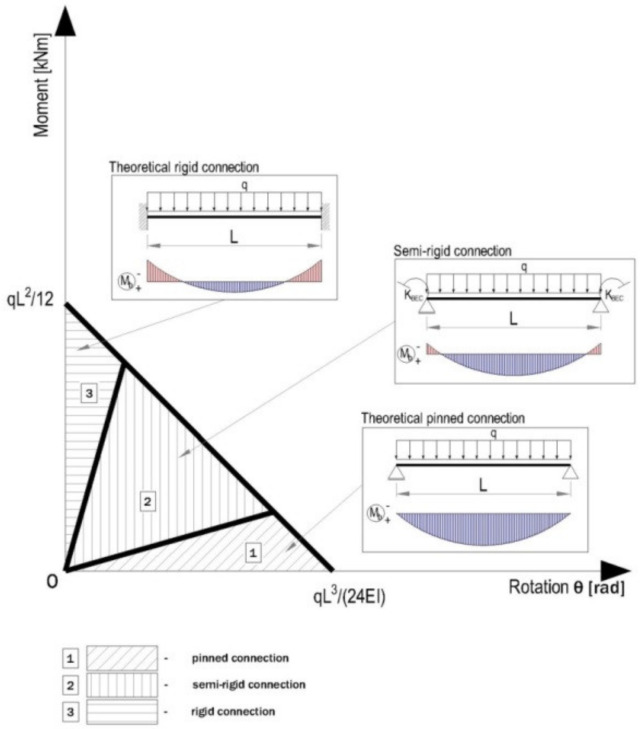
Classes of the beam-end-connections related to the rigidity, according to EN 1993-1-3 [8].

On the other hand, for the semi-rigid connections between the beam and upright region, the values of the rotation angle θ and of the bending moment M_b_ are between the values corresponding to the pin-connection and fixed support. Depending on the rigidity of the connection, in Figure 8, a classification of the connections is presented, according to EN 1993-1-3, Eurocode 3 [8].

For the beam connected with the uprights of the storage system, by using semi-rigid connections whose rigidity k_m_ was experimentally determined, the bending moment M_end_ developed at the ends of the beam subjected to the uniformly distributed force q, is computed by using Equation (15).

(15) $$M_{\mathrm{end}}=\frac{{\mathrm{qL}}^{3}}{24\mathrm{EI}\left(\frac{1}{k_{m}}+\frac{L}{2\mathrm{EI}}\right)}.$$

The bending moment M_mid_ developed at the middle cross section of the beam is computed using Equation (16).

(16) $$M_{\mathrm{mid}}=\frac{{\mathrm{qL}}^{2}}{8}-M_{\mathrm{end}}.$$

In order to analyze the capacity of the semi-rigid connections of the beam, it is computed by the safety coefficient c of the connection as the ratio between the design moment M_Rd_ computed by using Equation (12), based on experimental results, and the bending moment M_end_ developed at the end of the beam with semi-rigid connections at both ends and loaded with the uniformly distributed force q. The safety coefficient c of the connection is computed by Equation (17).

(17) $$c=\frac{M_{\mathrm{Rd}}}{M_{\mathrm{end}}}.$$

The value of the maximum deflection v_max_ having two semi-rigid connections at both ends, whose rigidity k_m_ was experimentally determined, is computed by using Equation (18).

(18) $$v_{\max}=\frac{{\mathrm{qL}}^{4}\left(10\mathrm{EI}+{\mathrm{Lk}}_{m}\right)}{384\mathrm{EI}\left(2\mathrm{EI}+{\mathrm{Lk}}_{m}\right)}.$$

Practically, customizing Equation (18) for the case of the beam pin-connected (hinged) at both ends for which the rigidity of the connection k_m_ is equal to zero, leads to the maximum deflection v_max_ equal with5qL^4^/ 384EI. On the other hand, customizing Equation (18) for the case of the beam fixed at both ends (embedded ends) for which the rigidity of the connection k_m_ tends to infinity, leads to the maximum deflection v_max_ equal toqL^4^/ 384EI.

## 3. Results

### 3.1. Tensile Properties of the Materials

In Table 5, the tensile properties corresponding to the tensile specimens cut from each component are shown. The tensile test results for the steel corresponding to the four-tab connector are identical with the ones corresponding to the five-tab connector for the same beam height because we used connectors with five tabs for all beams and we had cut the connector to obtain four-tab connectors. The decision regarding the cutting of the five-tab connector was taken in order to have the same material for both kinds of connectors.

### 3.2. Results of Bending Tests

In this research, all upright-connector-beam assemblies presented in Table 4 were tested according to the testing procedure presented in EN 15512 [9].

**Figure 9:**
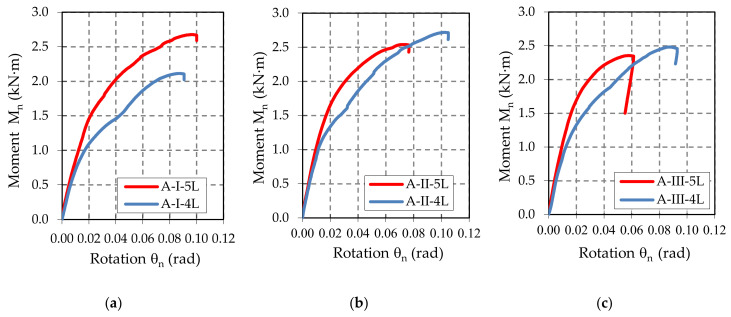
Comparison of the moment-rotation curves recorded for: (**a**) A-I-4L and A-I-5L assemblies, (**b**) A-II-4L and A-II-5L assemblies, and (**c**) A-III-4L and A-III-5L assemblies.

**Figure 10:**
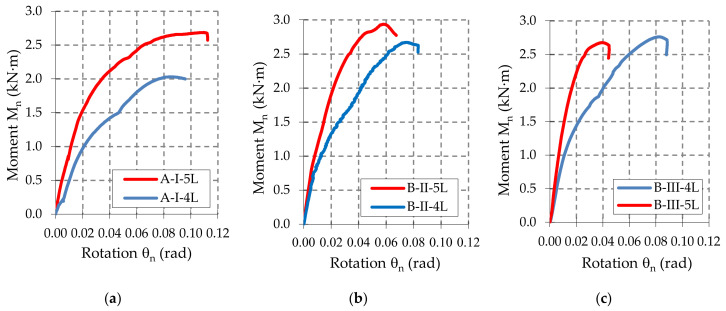
Comparison of the moment-rotation curves recorded for: (**a**) B-I-4L and B-I-5L assemblies, (**b**) B-II-4L and B-II-5L assemblies, and (**c**) B-III-4L and B-III-5L assemblies.

**Figure 11:**
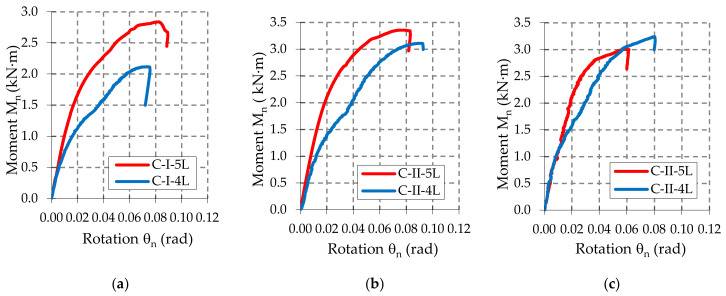
Comparison of the moment-rotation curves recorded for: (**a**) C-I-4L and C-I-5L assemblies, (**b**) C-II-4L and C-II-5L assemblies, and (**c**) C-III-4L and C-III-5L assemblies.

**Figure A1:**
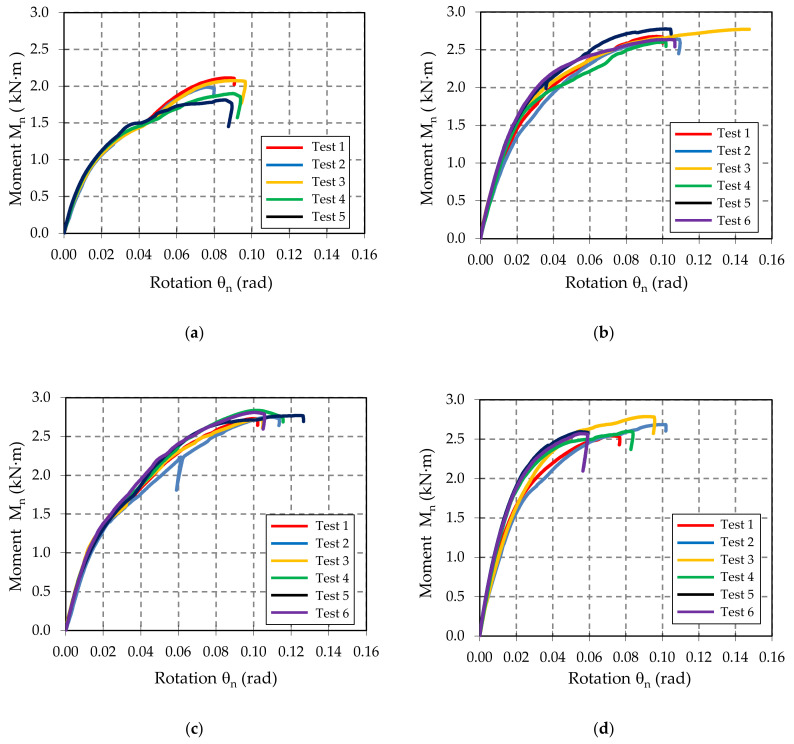

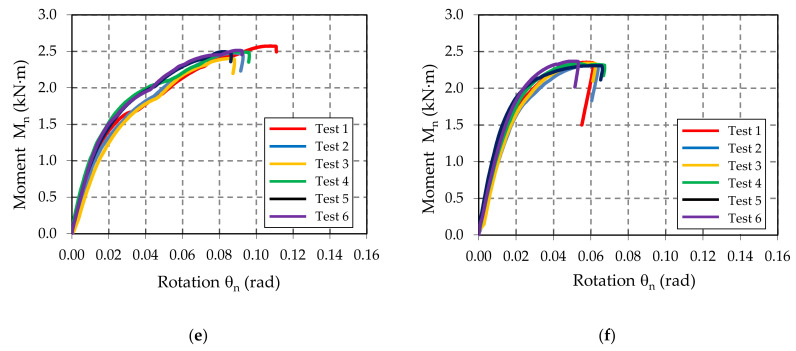
Moment-rotation (M_n_–θ_n_) curves recorded for the beam-connector-upright assemblies: (**a**) A-I-4L, (**b**) A-I-5L, (**c**) A-II-4L, (**d**)A-II-5L, (**e**) A-III-4L, and (**f**) A-III-5L.

**Figure A2:**
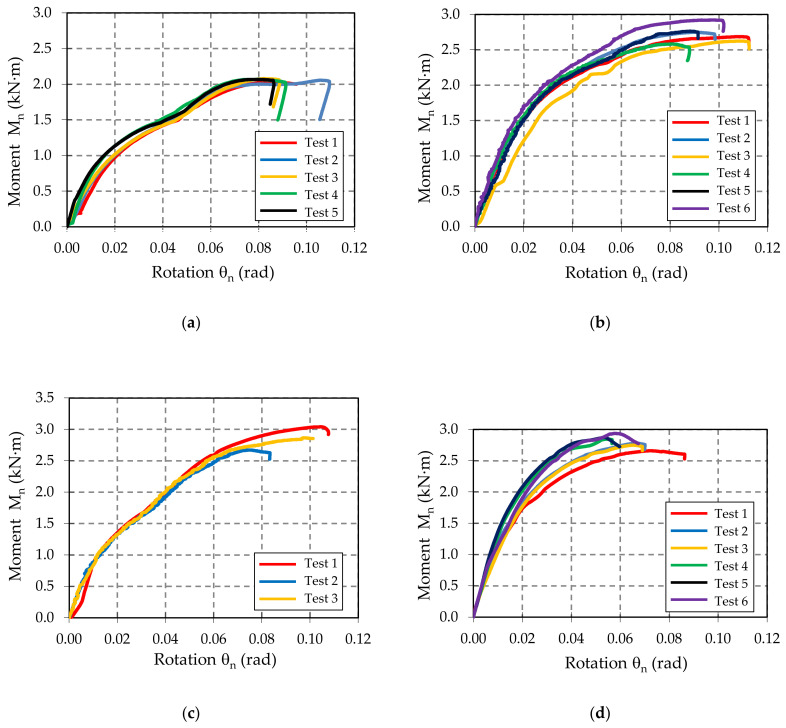

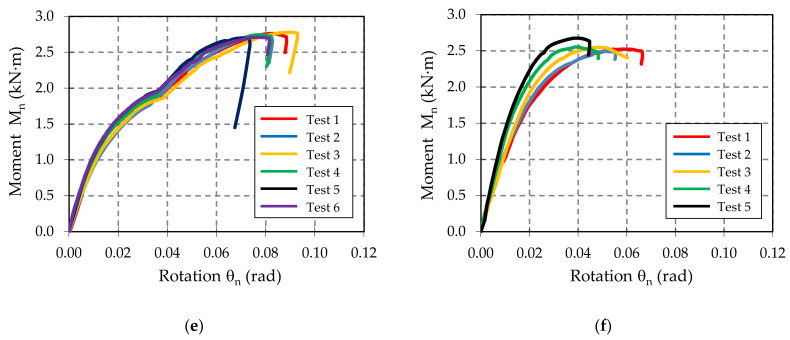
Moment-rotation (M_n_–θ_n_) curves recorded for the beam-connector-upright assemblies: (**a**) B-I-4L, (**b**) B-I-5L, (**c**) B-II-4L, (**d**) B-II-5L, (**e**) B-III-4L, and (**f**) B-III-5L.

**Figure A3:**
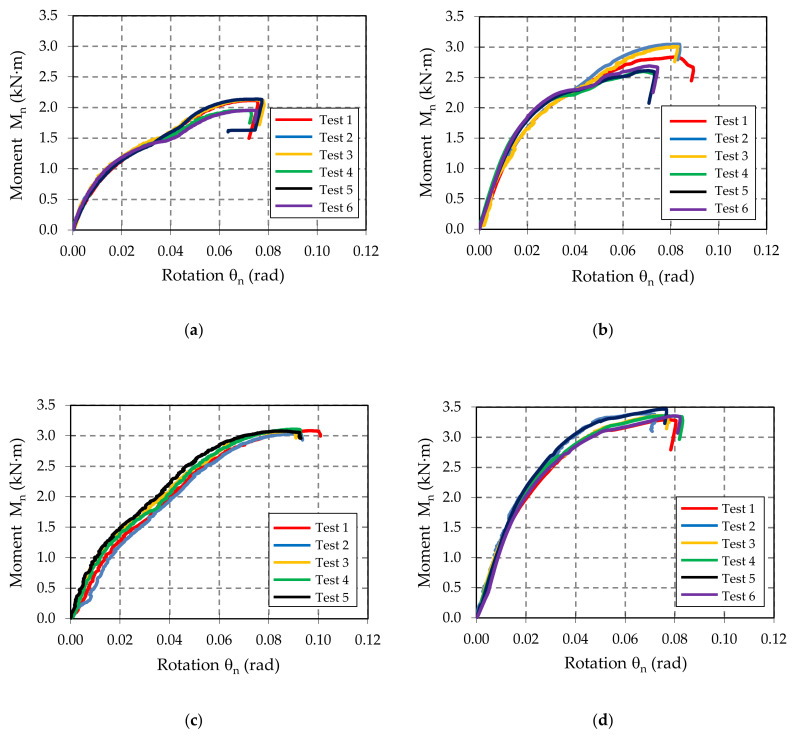

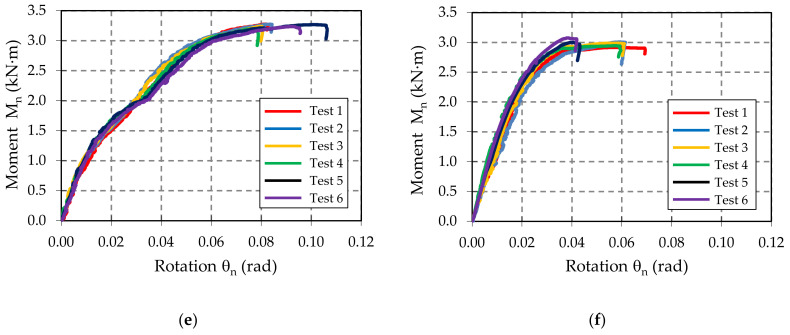
Moment-rotation (M_n_–θ_n_) curves recorded for the beam-connector-upright assemblies: (**a**) C-I-4L, (**b**) C-I-5L, (**c**) C-II-4L, (**d**) C-II-5L, (**e**) C-III-4L, and (**f**) C-III-5L.

First, the moment-rotation curves (M–θ) are represented by using the data acquired with the force and displacement transducers and by using Equations (1) and (2). Then, the corrected moment-rotation curves (M_n_–θ_n_) are plotted for each upright-connector-beam assembly, after the corrections caused by the different thickness and yield strength were made by using Equations (5) and (8). The moment-rotation curves (M_n_–θ_n_) corresponding to all sets of the assemblies are plotted in Appendix A. There are a total of 101 curves plotted. In Figure A1, it is shown that the curves for the beam-connector-upright assemblies containing a type A beam. In the same manner, in Figure A2 and Figure A3, the curves for the beam-connector-upright assemblies containing a type B beam and a type C beam, respectively. In order to comparatively analyze the effects of the connector type on the mechanical behavior of the beam-upright connections in bending the moment-rotation mean curves (M_n_–θ_n_) are plotted in Figure 9, Figure 10 and Figure 11. It may be observed that the behavior of the connections is nonlinear and the connectors with five tabs are always stiffer than the connectors with four tabs for all assemblies tested.

The shapes of the moment-rotation (M_n_–θ_n_) curves shown in Figure A1, Figure A2 and Figure A3 added in the Appendix A, show a good repeatability recorded for each set of beam-connector-upright assemblies tested, concerning both the initial slopes of these curves (which gives the rotational stiffness) and the maximum values of the capable bending moments.

All values for the design bending moment M_Rd,i_ and for the rotational stiffness k_ni_ (i = 1,n) are determined by bending tests of the upright-connector-beam assemblies and by using Equations (12) and (13), respectively. In order to make the comparisons for the test results, the stiffness k_ni_ was evaluated for the maximum bending moment M_Rd,i_ when η = 1. All results are presented in Table 6.

The average values and standard deviations are also shown in Table 6 for the design bending moment M_Rd_ and for the rotational stiffness k_m_ corresponding to each group of beam-connector-upright assemblies tested in bending. The value of the standard deviation corresponding to the design bending moment M_Rd_ for each group of assemblies, divided by the average value M_Rd_, is generally less than 3% and this means a high level of confidence of the experimental tests. Similar remarks may also be made regarding the values of the standard deviation corresponding to the rotational stiffness k_m_. The low values of stdev compared to the average values show that the degree of scattering of the results is small. The differences between the results obtained for assemblies of the same group are justified by the geometric imperfections of the cross sections, which are characteristic of the cold formed steel profiles, especially in the area of the slots of the upright profile.

**Figure 12:**
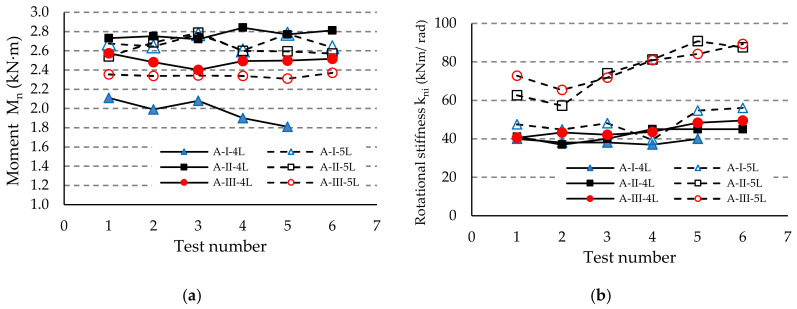
Comparison of the results obtained for the assemblies with the beam of type A concerning: (**a**) capable corrected moment M_n_, and (**b**) rotational stiffness k_ni_ of the connections.

**Figure 13:**
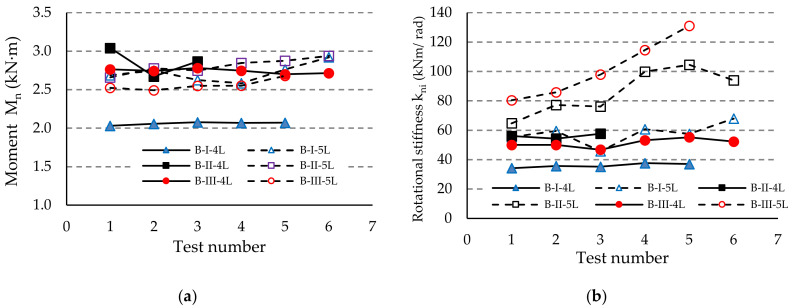
Comparison of the results obtained for the assemblies with the beam of type B concerning: (**a**) capable corrected moment M_n_ and (**b**) rotational stiffness k_ni_ of the connections.

**Figure 14:**
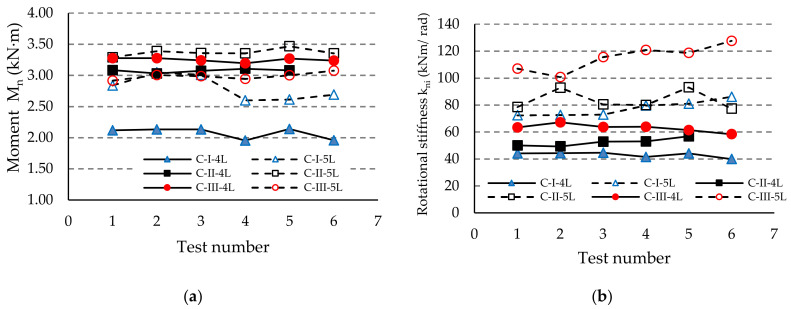
Comparison of the results obtained for the assemblies with the beam of type C concerning: (**a**) capable corrected moment M_n_, and (**b**) rotational stiffness k_ni_ of the connections.

The results regarding both the corrected moment M_ni_ and the rotational stiffness k_ni_ are comparatively shown in Figure 12, Figure 13 and Figure 14 for each type of beam used in the assembly, i.e., for beam A, beam B, or beam C, respectively. It notes that the degree of scattering of the results is small from the point-of-view of both the corrected moment M_ni_ and rotational stiffness k_ni_ corresponding to each group of assemblies tested (Figure 12, Figure 13 and Figure 14). Comparing the results corresponding to the assemblies composed by the same type of beam and upright, we noticed that the design rotational stiffness k_m_ increases as the upright thickness increases. It is also evident that the design rotational stiffness k_m_ is greater for the assembly containing the five-tab connector than the value corresponding to the assembly containing the four-tab connector (Table 6, Figure 12b, Figure 13b and Figure 14b).

Regarding the capable load, the design moment M_Rd_ corresponding to the assembly containing the upright II having a thickness of 1.75 mm is greater than the design moment M_Rd_ corresponding to the assembly containing the upright I with a thickness of 1.5 mm (Table 6, Figure 12a, Figure 13a and Figure 14a). However, the design moment M_Rd_ corresponding to the assembly containing the upright III with a thickness of 1.5 mm and the five-tab connector is less than the design moment M_Rd_ for the assembly with the same upright region and with four connectors, even if the stiffness is bigger for the assembly with a five-tab connector comparatively with the stiffness for the assembly with a four-tab connector. Moreover, the design moment M_Rd_ for the assembly containing upright III and a four-tab connector is much closer to the design moment M_Rd_ for the assembly containing upright II and a five-tab connector in the case of all types of beams (A, B, or C).

### 3.3. Failure Modes in Bending Tests

**Figure 15:**
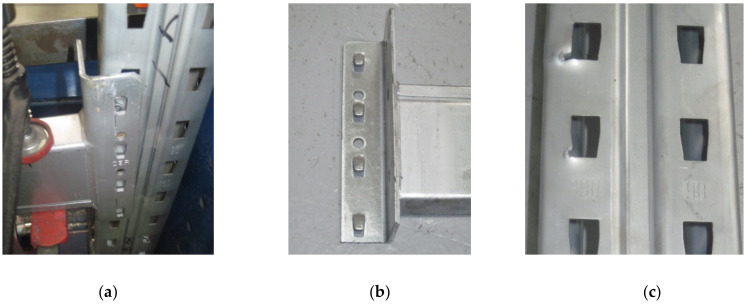
Failure modes of the upright region with a thickness equal to 1.50 mm: (**a,b**) failure mode of the connector and (**c**) failure mode of the upright region.

The failure mode was changed from assembly to another assembly tested. Upright is the main failure mode, especially when the upright thickness is 1.50 mm (Figure 15). This happens because, when the beam is loaded and the connector rotates, the first two tabs are tearing the upright’s slots because the upper part of the beam is subjected to tensile stresses (Figure 15c).

**Figure 16:**
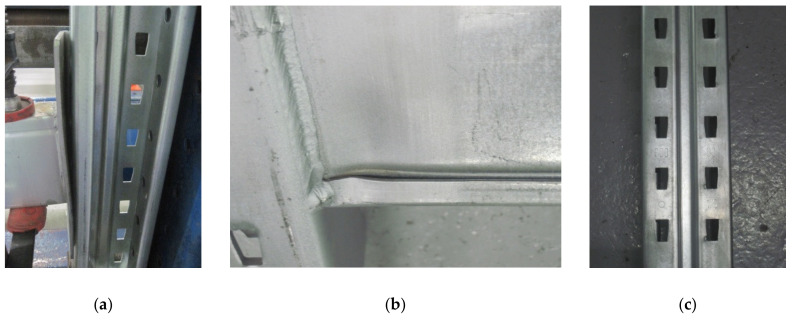
Failure modes in case of the assembly with an upright region having a thickness equal to 1.75 mm: (**a**) failure mode of the connector, (**b**) failure mode of the beam, and (**c**) failure mode of the upright region.

It was observed that, when the thickness of the upright increases, for the upright having a thickness of 1.75 mm, the failure mode was changed. All the elements (upright, beam, and beam end connector) start to be a part of the failure mode (Figure 16). In this case, the rotation angle θ deceases (Figure 16a), especially as the beam height increases and the bottom part of the beam is compressed and it starts to locally buckle (Figure 16b). The connector is also bending (Figure 16a).

**Figure 17:**
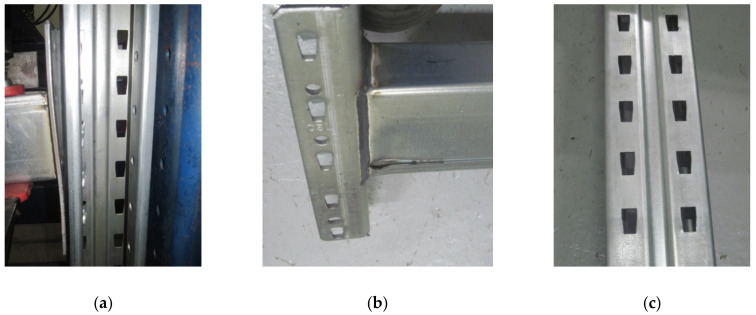
Failure modes in case of the assembly with the upright region having a thickness equal to 2.00 mm and a connector with five tabs: (**a**) failure mode of the connector, (**b**) failure mode of the beam, and (**c**) failure mode of the upright region.

**Figure 18:**
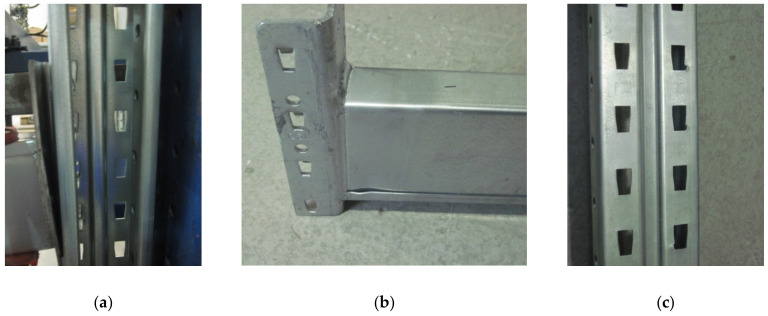
Failure modes in case of the assembly with an upright region having a thickness equal to 2.00 mm and a connector with four tabs: (**a**) failure mode of the connector, (**b**) failure mode of the beam, and (**c**) failure mode of the upright region.

For uprights having a thickness of 2.00 mm, the effects of the action of the connector tabs on the upright region is very small, especially for the five-tab connector (Figure 17a,c). The rotation of the connection is even smaller (Figure 17a), which means that the biggest rotational stiffness corresponds to this situation. The main failure modes were bending of the connector and the local buckle of the beam near the connector (Figure 17b). For assembly with a four-tab connector (Figure 18) because the beam rotates more than in a situation of the assembly with a five-tab connector, the first top tab still leaves a mark on the upright slots (Figure 18c).

## 4. Discussion

Analyzing the experimental results summarized in Table 6 and moment-rotation curves (M–θ) shown in Figure 9, Figure 10 and Figure 11, we note the following important findings.

- For the assemblies containing the uprights of type I with a thickness of 1.50 mm, the five-tab connector leads to a higher value of the design moment M_Rd_ and higher rotational stiffness k_m_ than in the assemblies with four-tab connectors. This behavior was noticed for all three types of beams (A, B, C) with different heights of the beam being cross sectional (Table 6).
- When the upright thickness increases (upright III), a change in the failure modes was observed because the five-tab connector rotates less than the four-tab connector (Figure 17 and Figure 18). The rotational stiffness k_m_ of the connection is higher for the assemblies with five-tab connectors than for the assemblies with four-tab connectors. On the contrary, the capable design moment M_Rd_ decreases for a five-tab connector, which is more visible in the bending tests applied to the assembly that contain the uprights of type III having a thickness of 2.00 mm (Table 6).
- As the upright thickness increases, the portion of the moment-rotation curves corresponding to the plastic behavior for the assemblies containing four-tab connectors is greater than the curve portion in plastic corresponding to the assemblies containing the five-tab connectors (Figure 9, Figure 10 and Figure 11). The mechanical behavior of the material is characteristic of elastic-plastic metallic materials [10,24].
- For the assemblies containing B or C beams with a section height of 100 mm or 110 mm, respectively, and containing a five-tab connector, the design moment M_Rd_ and rotational stiffness k_m_ of the joints increase due to the upright sections having a thickness of 1.5 mm or 1.75 mm. For the assemblies containing the upright sections with a thickness of 2.00 mm, the rotational stiffness k_m_ still increases, but design moment M_Rd_ of the connection decreases. In the assemblies with a 5-tab connector, the capable design moment M_Rd_ corresponding to the upright III having the thickness of 2.00 mm, is lower than the capable moment M_Rd_ corresponding to the upright I with a thickness of 1.75 mm upright regions in case of all types of beams.

In practice, the values of the design moment M_Rd_ and rotational stiffness of the connection experimentally obtained and given in Table 6 are very useful in order to check if the beam-connector-upright assembly works safely from both strength and stiffness points-of-view.

**Figure 19:**
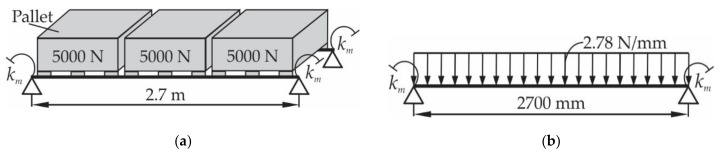
Scheme of loading: (**a**) loading for two beams, and (**b**) equivalent scheme of loading for calculus (k_m_ represents the rotational stiffness of the connections).

We considered a typical case for storage pallet racking systems for which two beams having the length of 2.7 m must support the weight of three wooden pallets (Figure 19a). The maximum total mass for one pallet loaded with goods is 500 kg per pallet, which means that the total weight is approximately 15,000 N for three pallets. This weight applied to one shelf is supported by two beams, which means approximately 7500N applied to the beam length of 2700 mm, which leads to a distributed force of 2.78 N/mm by assuming that the weight is uniformly distributed on the beam (Figure 19b). We considered that this beam is connected with upright regions by one type of semi-rigid connections tested for which the experimental results regarding the design moment M_Rd_ and rotational stiffness of the connection are synthesized in Table 6.

We aim to comparatively analyze the safety coefficient c for all beam-connector-upright assemblies tested to show in this way the reserve of each connection so that it works in the safety range. For this purpose, the moment developed at the end of the beam (at the level of beam end connection) is computed, which is denoted by M_end_ by using Equation (15) for the uniformly distributed force q of 2.78 N/mm applied to the beam with the length L of 2700 mm.

The safety coefficient c corresponding to each beam-connector-upright assembly involved in this study is computed by using Equation (17). Lastly, the results are synthesized and analyzed comparatively in Table 7 for all assemblies involved in this research. In Table 7, for each class of assemblies corresponding to one type of beam, the smallest and the highest value of the safety coefficient c is highlighted by using superscript symbols for those values (see the footnotes of Table 7).

In Table 7, we may remark that, for the assemblies containing types A or C beams, the best assemblies are based on the upright of type II and beam-end connector with four tabs (A-II-4L and C-II-4L in Table 7). Just in case of the beam of type B, the highest value of the safety coefficient c is for the assembly containing the upright of type III, which has the greatest thickness of 2 mm, and the beam-end connector with four tabs (B-III-4L in Table 7).

For each class of assemblies corresponding to a certain type of beam, the smallest safety coefficient c corresponds to the assembly for which the beam is connected with the upright of type III by using the beam-end connector with five tabs (A-III-5L, B-III-5L, and C-III-5L in Table 7).

It was very interesting to remark that the ratio between the highest safety coefficient and the smallest safety coefficient is equal to 1.68 (i.e., 4.41/ 2.63) for the assemblies containing the beam of type A, 1.69 (i.e., 4.57/ 2.70) for the assemblies containing the beam of type B, and 1.73 (i.e., 5.94/ 3.42) for the assemblies containing the beam of type C. The conclusion is that, for any type of beam involved in this research, there is a reserve of approximately 70% regarding the safety coefficient, which depends on the type of the upright and end-beam connector used.

On the other hand, the beam used in racking storage systems must obey another requirement: the allowable deflection v_all_ is equal to a maximum of 5% from the beam length, according to EN15512 [9]. This is called a stiffness condition. This means that the allowable deflection v_all_ must be 13.5 mm for the beam length of 2700 mm.

For the practical example considered, the maximum deflection v_max_ of the beam having two semi-rigid connections at both ends is computed by using Equation (18) for all assemblies involved in this research and the results are shown in Table 7. In order to check the stiffness condition, the ratio between the allowable deflection v_all_ of 13.5 mm and the maximum deflection v_max_ for the beam with the same semi-rigid connection at both ends is given for each type of beam-connector-upright assembly involved. It is remarked that all values computed for the v_all_/v_max_ ratio are greater than 1 and this means that the stiffness condition is obeyed for all assemblies. As expected, the ratio v_all_/v_max_ increases as the thickness for the beam or for the upright increases. In Table 7, it is observed that the best assemblies from the stiffness point-of-view (i.e., the greatest values of the ratio v_all_/v_max_) do not correspond to the best values for the safety coefficient c.

For better understanding the importance of the experimental determination of the connection’s rotational stiffness, a comparative study is presented for the same practical study case corresponding to the beam of type B for the following beam models.

**Figure 20:**
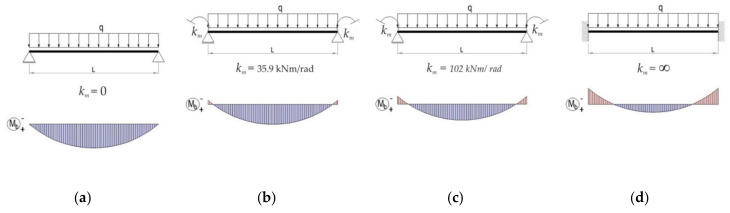
Bending moment diagram in case of different boundary conditions for beam B: (**a**) hinged beam ends, (**b**) semi-rigid connector with four tabs, (**c**) semi-rigid connector with five tabs, and (**d**) rigid connection (k_m_ represents the rotational stiffness of both end connections).

(i) beam B shown in Figure 20a, for which the rotational stiffness of the connections is equal to 0 that corresponds to hinged beam ends,
(ii) beam B shown in Figure 20b, for which the rotational stiffness k_m_ of both end connections is equal to 35.9 kNm/rad corresponding to the semi-rigid connection,
(iii) beam B shown in Figure 20c, for which the rotational stiffness k_m_ of both end connections is equal to 102 kNm/rad corresponding to the semi-rigid connection,
(iv) beam B shown in Figure 20d, for which the rotational stiffness k_m_ of the connections is infinite and corresponds to the rigid connections (fixed beam ends).

Furthermore, using RFEM software (Dlubal software Gmbh, Tiefenbach, Germany), it investigates the bending moment M_mid_ developed at the level of the cross section of the beam located at the midpoint of the beam, the bending moment M_end_ developed at the level of the connection, and the maximum deflection. Beam finite elements with two nodes were used in a numerical model of the beam loaded, as shown in Figure 19b. For finite element analysis (FEA) by using RFEM software, the beam corresponding to each case (Figure 20) was divided in 10 segments. Tridimensional support conditions were used in FEA at the beam ends in order to define the following boundary conditions for all cases: (i) beam hinged at both ends – all degrees of freedom are set to zero at supports, except the rotation with respect to the axis perpendicular to the loading plane (Figure 20a), (ii) beam with 4-tab connections at both ends—all degree of freedom are set to zero and the rotational stiffness k_m_ is set to 35.9 kN⋅m/rad at both end supports (Figure 20b), (iii) beam with 5-tab connections at both ends – all degrees of freedom are set to zero and the rotational stiffness k_m_ is set to 35.9 kN⋅m/rad at both end supports (Figure 20c), (iv) rigid connections (embedded) at both ends—all degrees of freedom are set to zero (Figure 20d). It is mentioned that all results are reported in case of the assemblies corresponding to the type B beam. In case of the beam models with semi-rigid connections at both ends (Figure 20b,c), only the cases corresponding to the extreme values of the rotational stiffness k_m_ are taken into account.

**Figure 21:**
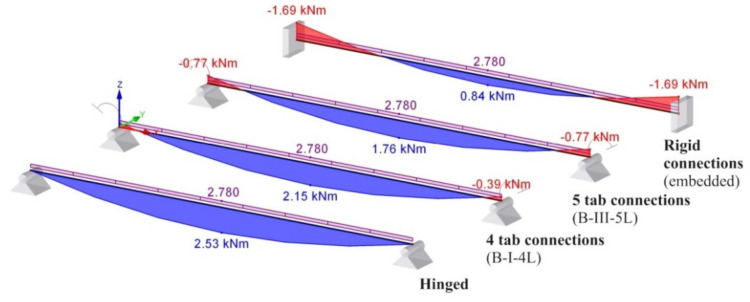
Comparison of the bending moment diagrams for different boundary conditions in case of the type B beam.

**Figure 22:**
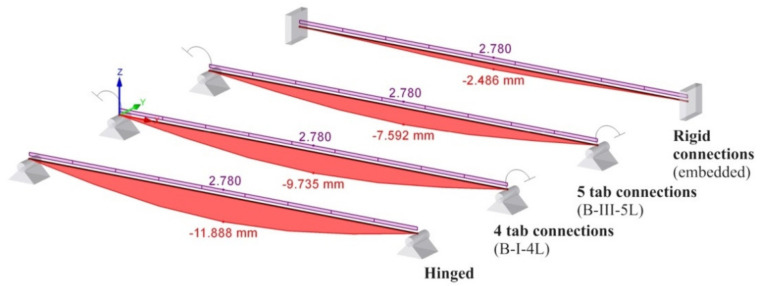
Comparison of the maximum deflection v_max_ for different boundary conditions in case of the type B beam.

**Figure 23:**
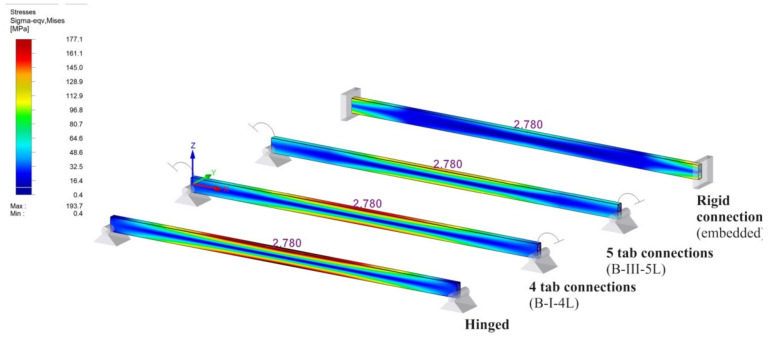
Comparison of the distribution of the equivalent stresses (von Mises) for different boundary conditions in case of the type B beam.

For better visual comparisons, the bending moment diagrams and deflection diagrams are graphically shown in Figure 21 and in Figure 22, respectively, for all cases shown in Figure 20. In the same manner, the distribution of the equivalent stress by Von Mises failure theory is comparatively plotted in Figure 23.

The results shown in Figure 21, Figure 22 and Figure 23, show that it is important to determine the stiffness of such semi-rigid connections because the deflection, the bending moment, and stresses are very much influenced by the boundary conditions.

## 5. Conclusions

Semi-rigid connections are often used in the industry of the pallet racking storage systems. This experimental study presents the mechanical behavior of the beam-end connectors used to assembly the beam with the upright regions. The main objective of this research was to investigate the influence of the type of the beam-end connectors (four-tab connector and five-tab connector) and also the effects of the thickness of the upright section wall on the capacity of the connections when the beam of the storage system is mechanically loaded. For all upright-connector-beam assemblies analyzed, the moment-rotation curves were plotted and the capable moment and the rotational stiffness of the connections are compared.

The research proved that, for the assemblies containing the uprights of type I having a thickness of 1.50 mm, the five-tab connector leads to a higher value of the design moment M_Rd_ and higher rotational stiffness k_m_ than in the assemblies with four-tab connectors. The rotational stiffness k_m_ is greater by approximately 23.1%, with 61% and with 77.1% for the assemblies containing five-tab connectors than for the assemblies containing five-tab connectors in case of type A, B, and C beams, respectively. The increase of the design moment M_Rd_ is approximately equal to 49.3%, 21.7%, and 26.5% for the assemblies containing five tab connectors than for the assemblies containing five-tab connectors in case of type A, B, and C beams, respectively.

On the contrary, for the assemblies containing the uprights of type III with a thickness of 2.00 mm, the capable design moment M_Rd_ decreases for the assemblies with five-tab connectors with respect to the values recorded for the four-tab connector. The decrease of the design moment M_Rd_ is indeed small, it is approximately equal to 3.2%, 10.3%, and 9.7% for the assemblies containing five-tab connectors than for the assemblies containing five-tab connectors in case of type A, B, and C beams, respectively.

It was shown that, for each class of assemblies corresponding to a certain type of beam, the highest value recorded for the rotational stiffness k_m_ obtained in bending tests of the connections, does not lead to the highest value of the safety coefficient c for that connection. Moreover, for a beam of type A and C, the best assemblies (A-II-4L and C-II-4L) from a safety coefficient point-of-view lead to the reduction of the mass of the racking storage system due to the thickness of the upright region and due to using the beam-end connector with four tabs. Additionally, this involves reducing material costs.

For the practical study case of the beam having the length of 2.7 m for which the same semi-rigid connector is used at both ends, the stiffness condition is obeyed, according to EN 15512 standard [9], for all types of beam-connector-upright assemblies involved in this research.

The experimental results concerning the rotational stiffness and the capable moment, obtained in this research are very important in modeling and simulation of the stresses and strain states in racking storage systems as long as the rotational stiffness of the beam-end connector is one of the input data in finite element analysis. In this context, the experimental results and test methods shown in this study can be used by the researchers who work in the field of the racking storage systems in order to obtain improved numerical models for such mechanical structures and good results by finite element analysis.

Some limitations of the research presented in this paper are related to the following aspects: (i) just one type of cross-section shape was considered for the upright profile, (ii) results obtained for both the rotational stiffness and design bending moment corresponding to the connections involved in this research, are valid for room temperature and are not valid in fire situations.

Taking into account the above limitations, there are some further directions of research identified. One of these research directions is to repeat the tests for similar groups of assemblies containing another type of thin-walled upright profile regarding the shape of the cross-section and, then, comparing the results with the ones presented in this paper in order to check if the effects of the upright thickness are the same. Another study could be made to make a numerical model or an analytical model in order to predict the rotational stiffness and the load-bearing capacity of the connections involved in this study for the accidental fire situations and to predict the time interval for maintaining the load-bearing capacity from the beginning of the fire.

## Figures and Tables

**Table 1:** Dimensions of the cross sections of the uprights.

Type of the Upright	Code of the Upright	D ^*^	W ^*^	B ^*^	T ^*^
		(mm)	(mm)	(mm)	(mm)
90 × 1.50	I	70	90	50	1.50
90 × 1.75	II				1.75
90 × 2.00	III				2.00

* Dimensions of the uprights are shown in Figure 2.

**Table 2:** Dimensions of the beams’ cross sections involved.

Beam Type	Beam Code	H ^*^	W ^*^	t ^*^
		(mm)	(mm)	(mm)
BOX 90	A	90	40	1.50
BOX 100	B	100	40	1.50
BOX 110	C	110	40	1.50

* Dimensions of the uprights are shown in Figure 3.

**Table 3:** Dimensions of the beam end connectors.

Connector Type	Connector Code	No. of Tabs	H ^*^	D ^*^	W ^*^	Thickness
			(mm)	(mm)	(mm)	(mm)
5-tab connector	5L	5	238	63	41	4.0
4-tab connector	4L	4	190	63	41	4.0

* Dimensions of the beam end connectors are shown in Figure 4.

**Table 4:** Upright-connector-beam assemblies tested.

Assembly Identification Code	Upright	Beam	Connector Type	Number of Tests for Assembly
A-I-4L	90 × 1.50	A (BOX 90)	4L	5
A-I-5L			5L	6
A-II-4L	90 × 1.75		4L	6
A-II-5L			5L	6
A-III-4L	90 × 2.00		4L	6
A-III-5L			5L	6
B-I-4L	90 × 1.50	B (BOX 100)	4L	5
B-I-5L			5L	6
B-II-4L	90 × 1.75		4L	6
B-II-5L			5L	6
B-III-4L	90 × 2.00		4L	3
B-III-5L			5L	5
C-I-4L	90 × 1.50	C (BOX 110)	4L	6
C-I-5L			5L	6
C-II-4L	90 × 1.75		4L	6
C-II-5L			5L	6
C-III-4L	90 × 2.00		4L	5
C-III-5L			5L	6

**Table 5:** Material properties for tested components.

Component Corresponding to the Tensile Specimen	Measured Thickness	Yield Stress Rp,02 f_t_ *	Ultimate Stress f_u_ *	Strain *
	(mm)	(MPa)	(MPa)	(%)
Upright I (90 × 1.50)	1.48	447.9	528.9	19.2
Upright II (90 × 1.75)	1.80	496.2	536.2	15.3
Upright III (90 × 2.00)	2.09	535.1	590.2	18.5
Beam A (box 90)	1.25	360.6	459.5	38.8
Beam B (box 100)	1.23	379.8	429.5	29.7
Beam C (box 110)	1.27	344.2	411.6	38.1
4-tab connector Box 90	4.04	409.9	469.8	25.6
5-tab connector Box 90	4.04	409.	469.8	25.6
4-tab connector Box 100	4.07	395.3	464	32.6
5-tab connector Box 100	4.07	395.3	464	32.6
4-tab connector Box 110	4.05	444.7	503.5	24.5
5-tab connector Box 110	4.05	444.7	503.5	24.5

* The values were obtained in tensile tests within this research carried-out by INSTRON 3369 machine.

**Table 6:** Test results in bending tests for all assemblies tested.

Assembly code	Design Moment			Rotational Stiffness			Assembly code	Design Moment			Rotational Stiffness			Assembly code	Design Moment			Rotational Stiffness		
	M_ni_	M_Rd_	Stdev	k_ni_	k_m_	Stdev		M_ni_	M_Rd_	Stdev	k_ni_	k_m_	Stdev		M_ni_	M_Rd_	Stdev	k_ni_	k_m_	Stdev
	(kNm)	(kNm)	(kNm)	(kNm/ rad)	(kNm/ rad)	(kNm/ rad)		(kNm)	(kNm)	(kNm)	(kNm / rad)	(kNm / rad)	(kNm / rad)		(kNm)	(kNm)	(kNm)	(kNm / rad)	(kNm / rad)	(kNm / rad)
**A-I-4L**	2.11	1.54	0.12	40	39.0	1.34	**B-I-4L**	2.03	1.84	0.02	34	35.9	1.38	**C-I-4L**	2.12	1.70	0.08	44	43.7	1.26
	1.99			38				2.06			36				2.13			44		
	2.08			38				2.08			35				2.13			45		
	1.90			37				2.07			38				1.95			41		
	1.81			40				2.07			37				2.14			44		
															1.96			40		
**A-I-5L**	2.68	2.3	0.07	47	48.0	6.18	**B-I-5L**	2.69	2.24	0.12	55	57.8	7.29	**C-I-5L**	2.84	2.15	0.20	72	77.4	5.71
	2.65			45				2.75			59				3.05			73		
	2.77			48				2.62			46				3.01			73		
	2.60			40				2.58			61				2.60			80		
	2.78			55				2.76			58				2.61			81		
	2.64			56				2.92			68				2.69			86		
**A-II-4L**	2.73	2.43	0.05	41	42.0	3.37	**B-II-4L**	3.04	2.03	0.18	56.1	56.0	1.65	**C-II-4L**	3.08	2.74	0.03	50.1	52.4	2.97
	2.75			37				2.67			54.3				3.03			49.3		
	2.72			40				2.87			57.6				3.07			52.8		
	2.84			45											3.11			53.1		
	2.77			45											3.08			56.8		
	2.81			45																
**A-II-5L**	2.54	2.21	0.09	63	76.0	13.5	**B-II-5L**	2.66	2.36	0.10	65	86.1	15.7	**C-II-5L**	3.29	2.95	0.06	79	83.7	7.25
	2.69			57				2.78			77				3.39			93		
	2.79			74				2.75			76				3.36			81		
	2.60			81				2.85			100				3.35			80		
	2.59			91				2.87			105				3.47			93		
	2.57			88				2.94			94				3.35			77		
**A-III-4L**	2.57	2.16	0.06	41	45.0	3.57	**B-III-4L**	2.76	2.43	0.03	50	51.2	2.97	**C-III-4L**	3.28	2.89	0.03	63	63.0	2.93
	2.48			43				2.74			50				3.27			67		
	2.40			42				2.78			47				3.24			64		
	2.49			44				2.75			53				3.20			64		
	2.50			48				2.70			55				3.27			61		
	2.52			50				2.71			52				3.24			58		
**A-III-5L**	2.36	2.09	0.02	73	77.0	8.90	**B-III-5L**	2.52	2.18	0.07	80	102	20.9	**C-III-5L**	2.92	2.61	0.06	107	115	9.76
	2.34			65				2.49			86				3.01			101		
	2.34			72				2.55			98				2.99			116		
	2.34			81				2.55			114				2.94			121		
	2.31			84				2.68			131				3.00			119		
	2.37			89											3.08			128		

**Table 7:** Results for the safety coefficient c of the connection for the maximum deflection in case of the assemblies tested for the case of the real loading in the pallet racking storage system.

Assembly Code	Rotational Stiffness k_m_	Design Moment M_Rd_	Moment at End M_end_	Safety Coefficient c	Maximum Deflection v_max_	Ratio v_all ***_/ v_max_
	(kN·m/rad)	(kN·m)	(kN·m)		(mm)	(%)
A-I-4L	39.0	1.54	0.52	2.94	12.35	1.09
A-I-5L	48.0	2.30	0.60	3.82	11.74	1.15
A-II-4L	42.0	2.43	0.55	4.41 ^*^	12.14	1.11
A-II-5L	76.0	2.21	0.79	2.80	10.29	1.31
A-III-4L	45.0	2.16	0.58	3.75	11.94	1.13
A-III-5L	77.0	2.09	0.79	2.63 ^**^	10.25	1.32
B-I-4L	35.9	1.84	0.41	4.47	10.31	1.31
B-I-5L	57.8	2.24	0.58	3.88	9.31	1.45
B-II-4L	56.0	2.03	0.57	3.59	9.38	1.44
B-II-5L	86.1	2.36	0.74	3.20	8.34	1.62
B-III-4L	51.2	2.43	0.53	4.57 ^*^	9.58	1.41
B-III-5L	102.0	2.18	0.81	2.70 ^**^	7.91	1.71
C-I-4L	43.7	1.70	0.40	4.22	8.27	1.63
C-I-5L	77.4	2.15	0.60	3.56	7.30	1.85
C-II-4L	52.4	2.74	0.46	5.94 ^*^	7.99	1.69
C-II-5L	83.7	2.95	0.63	4.65	7.15	1.89
C-III-4L	63.0	2.89	0.53	5.50	7.68	1.76
C-III-5L	115.0	2.61	0.76	3.42 ^**^	6.52	2.07

* The greatest safety coefficient of the connection for the group assemblies corresponding to one type of beam. ** The smallest safety coefficient of the connection for the group assemblies corresponding to one type of beam. *** The allowable deflection v_all_ is 13.5 mm for the beam length of 2700 mm, according to EN15512 [9].

## Appendix A

In Figure A1: the moment-rotation (M_n_–θ_n_) curves recorded for the beam-connector-upright assemblies containing type A beams in all tests carried out.

In Figure A2, the moment-rotation (M_n_–θ_n_) curves recorded for the beam-connector-upright assemblies are shown, which contain type B beams in all tests carried out.

In Figure A3, the moment-rotation (M_n_–θ_n_) curves recorded for the beam-connector-upright assemblies are shown, which contain type C beams in all tests carried out.
